# A better way of life: The role of leisure activities on self-perceived health, perceived stress, confidence in stress management, and satisfaction with social support in psychiatrists and psychiatry trainees in Mexico

**DOI:** 10.3389/fpsyt.2022.1052275

**Published:** 2022-12-07

**Authors:** Emmeline Lagunes-Córdoba, María Yoldi-Negrete, Tom Hewson, Diana Guízar-Sánchez, Rebeca Robles-García, Carlos Alfonso Tovilla-Zárate, Derek Tracy, Ricardo Arturo Saracco-Alvarez, Ana Fresán

**Affiliations:** ^1^Camden & Islington NHS Foundation Trust, London, United Kingdom; ^2^Subdirección de Investigaciones Clínicas, Instituto Nacional de Psiquiatría Ramón de la Fuente Muñíz, Mexico City, Mexico; ^3^Pennine Care NHS Foundation Trust, Ashton-under-Lyne, United Kingdom; ^4^Departamento de Fisiología de la Facultad de Medicina, Universidad Nacional Autónoma de México (UNAM), Mexico City, Mexico; ^5^Centro de Investigación en Salud Mental Global, Instituto Nacional de Psiquiatría Ramón de la Fuente Muñíz, Mexico City, Mexico; ^6^División Académica Multidisciplinaria de Comalcalco, Universidad Juárez Autónoma de Tabasco, Comalcalco, Tabasco, Mexico; ^7^West London NHS Trust, London, United Kingdom

**Keywords:** leisure activities, perceived stress, coping strategies, social support, psychiatrists

## Abstract

**Background:**

Psychiatrists are at high risk of developing burnout and mental health problems mainly due to their emotionally demanding jobs, difficult working conditions, long working hours, and poor work-life balance. As leisure activities are associated with better physical and mental health, engaging in these activities has been recommended as a measure to improve the wellbeing of healthcare workers. However, it is unclear the extent of which psychiatrists and trainees are involved in leisure activities, what type of activities they prefer, or how these impact their self-perceived health, stress, confidence in stress management, and satisfaction with their social support.

**Objective:**

The aim of this study was to identify differences in self-perceived health, perceived stress, confidence in stress management, and satisfaction with social support, between psychiatrists and trainees who engage in different leisure activities, compared with those who do not.

**Methods:**

This was a cross-sectional study, including Mexican psychiatrists (*n* = 355) and trainees (*n* = 330) who agreed to participate through an online survey.

**Results:**

73.1% of participants engaged in some leisure activity, being solitary-passive activities the most reported. Those who have a leisure activity reported lower stress, greater confidence in stress management, and more satisfaction with their social support. Passive-solitary activities were associated with less perceived stress and better confidence in stress management, while active-solitary and social activities were associated with better satisfaction with social support.

**Conclusion:**

Psychiatrists' and trainees' wellbeing benefits from engagement in leisure activities, which should be part of their daily schedules to reduce stress, and potentially improve their mental health.

## Introduction

Since the adoption of the biopsychosocial model in medicine and psychiatry, this model has received criticism regarding its actual use to understand, prevent and treat mental illness (1). However, the social aspect of this model has been recognized as a determinant for the onset and prevalence of both, physical and mental illness (2, 3). Although the study of social determinants has mainly focused on the impact of social inequality on people's health, other factors like working conditions, education, social support, and stress, have also been considered social factors influencing mental health and wellbeing that need to be addressed (4).

Like some social determinants, the impact of leisure activities on people's lives has been widely studied, and associated with better physical and mental health, life satisfaction, wellbeing, daily routine, and quality of life, at every stage of people's lives (5–7). Engaging in leisure activities has been associated with an increased sense of purpose and meaning, as well as serving as distractions from everyday worries. Similarly, leisure activities have been associated with better mood, and lower levels of stress, anxiety, and depression (8–10). Understandably, leisure covers a very broad list of activities, often defined as a voluntary, non-work-related activities that people engage with for enjoyment (5), with no formal consensus for their classification. However, some have classified them as physical or non-physical, individual or social, artistic or sports-related, and indoors or outdoors.

How leisure activities impact people's wellbeing has been attributed to different factors, including: increased physical activity, development of social relationships and positive emotions, acquisition of skills and knowledge, promotion of self-expression and creativity, and feelings of self-efficacy and self-determination (11, 12). Although there is no unifying framework explaining how leisure activities influence people's health and wellbeing, a recent review identified over 600 potential mechanisms of action (5), grouped into five interlinking and non-exclusive categories: (1) psychological processes, including building resilience and psychological capabilities, and developing a sense of self; (2) biological processes, involving modulation of the endocrine, immune, and nervous systems, and improvement of the cardiometabolic system and physical performance; (3) social processes, related to the improvement of social relationships, social resources and social identity, and the support of group cohesion and integration; (4) behavioral processes, such as promoting the development of habits and behavioral activation, increasing motivation and social responsibility; and (5) health behaviors, involving leisure activities encouraging disengagement from unhealthy ones (5). Clearly, some of these factors could be considered positive social determinants of health.

Although the benefits of engaging in leisure activities to improve people's health and wellbeing have been well-established before, there might be some barriers and limitations for people to be able to engage in these types of activities, barriers which could also be associated with social determinants, such as working conditions and stress. For example, people working long hours and in very demanding jobs, like healthcare professionals, might be less likely to have time to pursue leisure activities, and benefit from them. Nevertheless, even in these demanding scenarios, the benefits stand, as a recent study conducted in China identified that people who worked over 60 h a week, had a higher prevalence of poor mental health compared with those working <40 h; however, those who had a hobby were less likely to have poor mental health, even if working long hours (13).

It is well-known that healthcare workers, particularly doctors and trainees, are at high risk of developing burnout and mental health problems (14, 15), mainly due to their demanding jobs, long working hours, and poor work-life balance (16). Understandably, poor wellbeing and high levels of burnout in doctors have been associated with poor patient safety outcomes, as burnout doctors are more likely to underperform or make mistakes that can impact patients' care and satisfaction (17, 18). Therefore, despite time limitations, engaging in leisure activities might still be advisable to help reduce burnout and improve the wellbeing of healthcare workers, mainly during a time when the mental health and wellbeing of these professionals have been finally recognized as a priority for healthcare organizations (19); and addressing social determinants has also been considered essential to improve people's health.

Although it can be argued that most doctors, regardless of their specialty, are at risk of developing burnout at some point in their lives, psychiatrists require special attention, as psychiatrists have been found to be at higher risk of suicide compared with other specialists (20). A study conducted in Mexico showed that up to 7.6% of psychiatrists have experienced suicidal ideation at some point in their professional life (21). This increased suicidal risk could be associated not only with the characteristic of their profession but also with the Mexican context, as social determinants, like the shortage of physicians, increased workload, infrastructure constraints and income disparities, (22) negatively impact the mental health of these specialists.

Even though there is no doubt that engaging in leisure activities is likely to help psychiatrists and psychiatry trainees improve their wellbeing, it is unclear how many of them are involved in any leisure activity, what type of activities they prefer, or how these impact their perceptions of health, stress, their confidence in stress management or their satisfaction with social support. To address this, the present study aimed to identify self-perceived health, current perceived stress, confidence in stress management, and satisfaction with social support among psychiatrists and psychiatry trainees who have leisure activities compared with those who do not have such activities. We also aimed to compare how different types of leisure activities would impact these variables. The main hypotheses were that mental health professionals with leisure activities would show: (1) better self-perceived health, (2) lower perceived stress, (3) higher confidence in stress management and (4) higher social support than those without leisure activities, despite social constraints such as long working hours.

## Materials and methods

This was a cross-sectional study of Mexican psychiatrists and psychiatry trainees who voluntarily accepted to participate through an online survey.

### Participants

Our target population were all psychiatrists and psychiatry trainees working in Mexico registered in the Comprehensive System of the Division of Postgraduate Studies of the National Autonomous University of Mexico (UNAM, by the acronym of its name in Spanish: *Universidad Nacional Autónoma de México*) and the Interinstitutional Commission for the Training of Human Resources for Health. A total of 4,393 psychiatrists and 572 psychiatry trainees were eligible to participate at the time of the study. Participants were recruited using convenience sampling. Psychiatrists and trainees known to the researchers were invited to disseminate the survey within their networks to identify further eligible participants. Social media was also used to disseminate the link for the online survey. Furthermore, to increase recruitment, the Postgraduate Unit of UNAM supported researchers by contacting possible candidates by email to disseminate the survey. Recruitment was carried out from January 2018 to December 2018. The online survey included information regarding the nature and procedure of the study, including its anonymity, which was guaranteed to all participants. Those who accepted to participate in the study proceeded to complete the survey.

To capture survey responses we used RSForm!Pro in a Joomla! based website (both under GNU General Public License). The online survey was accessible only through a link which directed participants to the survey and was not available for general visitors of the site. The website is run by a non-profit organization focused on the dissemination of information regarding psychiatry. There were around 11 questionnaire items per page, with 15 pages in total, however, for the present study only 28 items were analyzed (detailed in the assessment procedure). Completeness checks were performed through JAVAScripts for mandatory items which were signaled to the users throughout the survey, and submission was not possible unless all mandatory items had been answered. A back button allowed responders to verify and change their answers if desired before submitting their survey. To identify duplicate entries, a combination of IP address and birthdate was used and when found, only the most recent entry was retained. We did not use cookies. Information regarding the nature and procedure of the study, as well as its anonymity which was guaranteed to participants, was included on the first page of the survey. Those who accepted to participate in the study proceeded to complete the survey. If a participant wanted to retire from the study, the participant just did not complete the questionnaire. Therefore, only completed surveys were analyzed (see Appendix).

### Assessment procedure

The survey was conducted in Spanish and took ~20 min to answer. It comprised six major sections: (1) Demographics and work-related information, including age, sex, marital status, having children, professional grade (psychiatrist or psychiatry trainees) and maximum working hours per day (excluding the continuous 36-h schedule of medical on-call shifts); (2) Self-perceived health, in which participants were asked to evaluate their current self-perceived health on a 100-point visual analog scale (0 = the worst-perceived state of health to 100 = the best-perceived state of health); (3) Perceived stress, in which participants were asked to rate their current level of stress on a scale from 0 to 100 (0 = not having stress to 100 = the maximum perceived stress); (4) Confidence in stress management, which was assessed with the validated Perceived Self-Efficacy Scale for Coping with Stress (23). This scale includes 8-items that assess efficacy and outcome expectations, scored on a Likert- agreement scale (1 = totally disagree to 5 = totally agree), with some items scored inversely, with higher scores reflecting better stress management. For the present study, we used the total score of the scale (Cronbach's alpha = 0.75) obtained by the sum of the two dimension's scores, with higher scores reflecting greater confidence in stress management. (5) Satisfaction with social support, which was evaluated with the Satisfaction dimension of the validated Spanish version of the Social Support Questionnaire (SSQ-6). This dimension includes 12 self-report items which assess the perceived adequacy of the social support received, for which items are rated on a Likert satisfaction scale from 1 = very dissatisfied to 6 = very satisfied. For this scale, average scores are used to establish levels of satisfaction with social support, with higher scores reflecting more social satisfaction (24). Reliability of the SSQ-6 in the Mexican population was adequate, with a Cronbach's alpha value of 0.85 (25). (6) Finally, leisure activities were assessed with a binary response question (no/yes) “*Do you have any leisure activity?”*. For those who gave an affirmative answer, a second open-ended question was added: “*Which ones?”*. The answers provided by participants were coded into three categories: (1) passive-solitary activities (e.g., pleasure reading, watching series and movies at home, listening to music, surfing the internet, etc.), (2) active-solitary activities (e.g., home activities as caring for pets, crafting, knitting; and artistic activities such as playing a musical instrument, painting, sculpture, writing, etc.) and (3) social activities (e.g., sports activities as going to the gym, play soccer, practicing yoga, CrossFit, and social interactions such as spending time with family or friends, going to the movies, theater or museums, traveling, etc.). Each participant could mention more than one type of leisure activity. For the present study four main groups were formed: (1) those without any leisure activity, (2) those who reported passive-solitary activities, (3) those with active-solitary activities and (4) those with social activities.

### Statistical analyses

Frequencies and percentages were used to describe categorical variables, means and standard deviations (S.D.) for continuous variables. Variables exhibited acceptable values of skewness (range −0.07 to 1.5) and kurtosis (−2.0 to 1.5); therefore, Chi-square tests (χ^2^) and one-way ANOVA with Bonferroni correction were used to compare demographic and work-related information, self-perceived health, current perceived stress, confidence in stress management and satisfaction with social support among psychiatrists and psychiatry trainees, based on the leisure activities groups previously defined. Group comparisons were performed in the following way: (1) without leisure activities vs. other activity vs. passive solitary activity; (2) without leisure activity vs. other activity vs. active solitary activity; (3) without leisure activity vs, other activity vs. social activity. Linear regression models with the backward method were performed to determine the influence of leisure activities on self-perceived health, perceived stress levels, confidence in stress management, and satisfaction with social support, which were defined as the outcome variables. From demographic data, sex, having children, being a psychiatry trainee or specialist, and the maximum current working hours per day were included in each model as we considered they could also affect the outcome variables in addition to the presence or absence of leisure activities. β-coefficients were used as effect size indicators for those variables with the highest association with each of the outcome variables. The alpha value for tests was set at *p* ≤ 0.05. We used the SPSS V.21 for Windows for the analysis.

## Results

A total of 685 psychiatry trainees (48.2%, *n* = 330) and psychiatrists (51.8%, *n* = 355) from 27 of the 32 states in Mexico completed the online survey (response rate for psychiatry trainees = 57.6% and 8.1% for psychiatrists according to the registers of the Comprehensive System of the Division of Postgraduate Studies of UNAM and the Interinstitutional Commission for the Training of Human Resources for Health). Women represented 51.8% (*n* = 355) of the sample, while the mean age of the sample was 35.5 (S.D.= 10.7) years old. A higher percentage of participants were single at the time of the study (67.0%, *n* = 459) and only 29.2% (*n* = 200) of participants reported having children. From the psychiatry trainees' group, 36.1% (*n* = 119) were in their first year of training, 28.8% (*n* = 95) in their second year, 29.4% (*n* = 97) in their third year and 5.8% (*n* = 19) in their fourth year. The maximum working hours reported were 12.8 (S.D. = 5.4) hours per day.

Regarding leisure activities, passive-solitary activities were the most frequently reported (43.5%, *n* = 298), followed by social activities (23.8%, *n* = 163) and active-solitary activities (19.4%, *n* = 133). The comparison of demographic features among participants who reported having leisure activities and those who did not are shown in Table 1. Fewer women reported having leisure activities compared with their male counterparts; and of those women who reported leisure activities, solitary active and social activities were reported more frequently than solitary passive activities, which were more frequent in men. Participants without any leisure activity were younger compared to the other groups, while participants who engaged in social activities were older than those who engaged in solitary activities. Participants with children were less likely to engage in any leisure activity; however, social activities were more frequently reported in those having children than any other type of activity. Participants who performed passive-solitary activities reported more working hours per day than those who did not report any leisure activity.

**Table 1:** Bivariate comparisons of demographics, work-related features, self-perceived health, perceived stress, confidence in stress management, and satisfaction with social support according to leisure activity type.

**Variables**	**Without activities (WA)**	**Solitary-passive activities (SP)**		**Solitary-active activities (SA)**		**Social activities (S)**		**Statistic**
		**No^a^**	**Yes**	**No^a^**	**Yes**	**No^a^**	**Yes**	
	***n =* 184**	***n = 203***	***n = 298***	***n = 368***	***n = 133***	***n = 338***	***n =* 163**	
**Demographics and laboral features**								
Sex (%)								SP χ^2^ = 20.9, *p* < 0.001 SA χ^2^ = 20.2, *p* < 0.001 S χ^2^ = 18.5, *p* < 0.001
Men	35.3	47.3	56.7	55.4	45.9	55.0	48.5	
Women	64.7	52.7	43.3	44.6	54.1	45.0	51.5	
Age (mean, S.D.)	32.9 (8.0)	36.8 (11.1)	36.1 (11.6)	36.4 (11.6)	36.5 (11.0)	35.4 (10.8)	38.5 (12.3)	SP *F* = 7.3, *p* = 0.001^b^ SA *F* = 7.1, *p* = 0.001^b^ S *F* = 12.0, *p* < 0.001^c^
Marital status (%)								SP χ^2^ = 1.6, *p* = 0.44 SA χ^2^ = 1.1, *p* = 0.55 S χ^2^ = 2.7, *p* = 0.25
Single	70.1	64.0	67.1	65.5	66.9	67.8	62.0	
Partnered	29.9	36.0	32.9	34.5	33.1	32.2	38.0	
Children (%)								SP χ^2^ = 8.9, *p* = 0.01 SA χ^2^ = 4.9, *p* = 0.08 S χ^2^ = 10.8, *p* = 0.004
No	77.2	63.5	71.8	68.2	69.2	71.9	61.3	
Yes	22.8	36.5	28.2	31.8	30.8	28.1	38.7	
Laboral status (%)								SP χ^2^ = 3.4, *p* = 0.17 SA χ^2^ = 2.1, *p* = 0.34 S χ^2^ = 9.0, *p* = 0.01
Trainee	52.7	43.3	48.7	46.7	45.9	50.6	38.0	
Specialist	47.3	56.7	51.3	53.3	54.1	49.4	62.0	
Hours of work (mean, S.D.)	12.3 (5.2)	12.2 (4.8)	13.5 (5.8)	12.9 (5.4)	13.1 (5.8)	13.3 (5.6)	12.4 (5.0)	SP *F* = 7.3, *p* = 0.01^d^ SA *F* = 7.1, *p* = 0.35 S *F* = 2.5, *p* = 0.07
**Self-perceived health**								
Self-perceived health (mean, S.D.)	67.7 (27.1)	68.5 (28.0)	69.8 (25.5)	68.2 (26.8)	72.4 (25.7)	69.7 (26.2)	68.5 (27.4)	SP *F* = 0.3, *p* = 0.69 SA *F* = 1.4, *p* = 0.22 S *F* = 0.3, *p* = 0.69
**Perceived stress and confidence in stress management**								
Perceived stress (mean, S.D.)	52.2 (29.5)	48.2 (29.0)	46.1 (29.1)	46.7 (29.2)	47.7 (28.5)	47.0 (28.7)	46.8 (29.8)	SP *F* = 2.4, *p* = 0.08 SA *F* = 2.1, *p* = 0.11 S *F* = 2.1, *p* = 0.12
Perceived self-efficacy scale for coping with stress (mean, S.D.)	24.1 (4.1)	25.6 (4.4)	25.7 (4.1)	25.7 (4.2)	25.6 (4.4)	25.6 (4.2)	25.8 (4.3)	SP *F* = 9.2, *p* < 0.001^b^ SA *F* = 9.2, *p* < 0.001^b^ S *F* = 9.2, *p* < 0.001^b^
**Satisfaction with social support**								
SSQ satisfaction score (mean, S.D.)	4.5 (1.5)	5.0 (1.3)	4.8 (1.3)	4.8 (1.3)	4.9 (1.3)	4.8 (1.4)	5.1 (1.1)	SP *F* = 6.4, *p* = 0.002^b^ SA *F* = 5.0, *p* = 0.007^b^ S *F* = 7.6, *p* = 0.001^b^

All comparisons were performed between participants who do not perform any leisure activity, those who reported a specific leisure activity, and participants who do not report the specific activity but reported other leisure activity.

a Participants reported performing other leisure activity.

b Bonferroni: WA vs. No (<0.05); WA vs. Yes (<0.05).

c Bonferroni: WA vs. No (<0.05); WA vs. Yes (<0.05); No vs. Yes (<0.05).

d Bonferroni: No vs. Yes (<0.05).

### Self-perceived health, perceived stress, confidence in stress management and satisfaction with social support according to reported leisure activities

As shown in Table 1, self-perceived health was similar between all groups. There were also no differences in perceived stress among the different types of leisure activities reported; however, when this variable was compared exclusively between those who reported any leisure activity and those who did not report any, those with any leisure activity had lower perceived stress levels (47.0, S.D. = 29.0 vs. 5.0, S.D. = 52.0, *t* = 1.9, *p* = 0.04). Similarly, levels of confidence in stress management were lower in participants who did not report performing any leisure activity than those who did. Finally, participants with leisure activities, particularly those who engaged in social activities, also had greater satisfaction with their social support, compared with those without any leisure activity.

### Influence of leisure activities on self-perceived health, perceived stress, confidence in stress management, and satisfaction with social support—Final linear regression models

The final regression models, shown in Table 2, showed that leisure activities had a specific influence on the outcomes assessed in the present study and that other confounders had a higher impact on them, particularly being a psychiatry trainee, which was associated with higher perceived stress and less confidence in one's abilities to deal with it; while being a graduated psychiatrist was associated with a better health perception and higher satisfaction with social support. The performance of passive-solitary leisure activities did not have an influence on self-perceived health or satisfaction with social support. However, these activities reduced the perception of stress and increased confidence in stress management. On the contrary, having active-solitary leisure activities and social leisure activities increased satisfaction with social support.

**Table 2:** Influence of leisure activities on self-perceived health, perceived stress, confidence in stress management and satisfaction with social support—final linear regression models.

	**Self-perceived health**		**Perceived stress**		**Confidence in stress management**		**Satisfaction with social support**	
	**β coefficient**	**95% C.I**.	**β coefficient**	**95% C.I**.	**β coefficient**	**95% C.I**.	**β coefficient**	**95% C.I**.
Sex	–	–	–	–	–	–	–	–
Having children	–	–	−2.2^c^	−12.1 to −0.9	2.2^a^	1.5–3.0	–	–
Professional grade—psychiatrist	15.0^a^	11.3–18.8	−6.0^c^	−11.1 to −0.9	1.6^a^	1.5–3.0	0.2^b^	0.07–0.5
Maximum hours of work per day	0.9^a^	0.6–1.3	–	–	0.05 ^c^	0.04–0.1	–	–
Passive-solitary leisure activity	–	–	−4.1^c^	−8.1 to −0.9	0.7^c^	0.1–1.3	–	–
Active-solitary leisure activity	–	–	–	–	–	–	0.2^c^	0.003–0.5
Social leisure activity	–	–	–	–	–	–	0.4^b^	0.1–0.6

a p ≤ 0.001.

b p ≤ 0.01.

c p ≤ 0.05.

Hyphen (–) indicates variables that did not enter in the regression model.

## Discussion

### Key findings

The results of this study showed that, despite their demanding jobs and long working hours, a large percentage of Mexican psychiatrists and psychiatry trainees (73.4%) engaged in some form of leisure activity, being solitary-passive activities, such as reading and engaging in artistic activities, the most common types of activities reported. Our results also showed that women, younger people, and people with children were less likely to have leisure activities compared with men, older participants and those who reported not having children. Regarding the influence of leisure activities on people's wellbeing, we identified that even though there were no differences in the levels of self-perceived health among people with or without leisure activities, participants who reported engaging in these, had lower stress levels, felt better equipped to manage their stress and had more satisfaction with their social support, compared with those who did not engage in leisure activities.

### Comparison with the literature

Although we could not identify studies comparing engagement with leisure activities in a similar population, those in broader populations have found that younger people and women tend to engage less in leisure activities, compared with older people and men (11, 26, 27), which is also supported by our results. Similarly, a study focused on Brazilian medical students, also found that female students were less likely to have leisure activities, compared with male students (28). Regarding the most common type of leisure activities identified in this study, our results are in line with what has been reported elsewhere, as other authors have also found solitary-passive activities (including listening to music and watching TV) to be the most common type of activities reported by physicians in the US and the UK (29, 30). However, these same studies also found doctors to be more involved in physical activities, with up to 50% of their sample reporting that they enjoy jogging or playing a sport, whereas only 19.4% of our participants reported engaging in active leisure activities. It is unclear why Mexican psychiatrists seem to be less active compared with doctors in the US and the UK. However, this might be associated with contextual or cultural factors, as a recent national survey showed that in Mexico, only 38.9% of the population reported engaging in regular physical activity, citing lack of time as the main reason for not exercising (31). Additionally, in Mexico there is a shortage of psychiatrists, with 3.7 psychiatrists per 100,000 inhabitants, which could translate into an important workload for both, psychiatrists and trainees, limiting their time available to engage in any psychical activity (32). According to our results, Mexican psychiatrists seem to be missing the potential health benefits of being more physically active, which could be an effective intervention to improve their physical health and wellbeing (33).

Despite the limited engagement in physical activities, which in our study, was associated with better satisfaction with social support, participants who engaged in social leisure activities were also more satisfied with their social support, compared with those who did not engage in any activity. This shows how different types of leisure activities could still have similar positive influences on people's wellbeing. Although leisure activities have been associated with the development of social relationships and improved connections (5, 12), most participants expressed being satisfied with their social support; so, this might also explain their limited engagement in leisure social activities. Having said this, satisfaction with social support was higher amongst participants who reported engaging in leisure activities than in those who did not. It is possible that psychiatrists and trainees with leisure activities might have benefited from building social identity, group cohesion and integration, elements, which have been identified as positive outcomes of having a leisure activity (5), hence, feeling more satisfied with the social support they already have.

Regarding self-perceived health, and opposite to what we expected, there were no differences between participants with and without leisure activities. One study comparing job satisfaction among Norwegian doctors, found that psychiatrists had higher levels of life and job satisfaction compared with other specialists, being only behind those working in public health (34). A study conducted in the US also found psychiatrists to be less likely to report burnout and to be more satisfied with work-life balance, compared to other specialists (35). Therefore, it is likely that the moderate self-perceived health reported by most participants, regardless of engagement in leisure activities, could be associated with the characteristics of the profession, which even when it can be considered psychologically demanding, it might also be more gratifying and less physically demanding than other medical specialities. However, this should be further assessed in other studies.

According to our results, there were moderate levels of perceived stress among most participants; however, those with leisure activities, mainly those who engaged in passive-solitary activities not only had lower levels of perceived stress, but they also felt better equipped to manage their stress, compared with those without any leisure activity. This is consistent with what has been reported in other studies, which have identified lower stress levels and improved health behavior in people who engage in leisure activities compared with those who do not (9, 10, 36). This suggests that engaging in leisure activities could not only help reduce stress in mental health professionals but also help them improve their confidence in their ability to manage stress.

### Limitations and strengths

There are some limitations to this study that need to be acknowledged. First, the use of a convenience sample approach, the lack of sample calculation, and the low response rate of psychiatrists compared to that of trainees, limit the generalization of our results to all psychiatrists in Mexico, therefore, results should be taken with caution. Second, as participants were asked if they had a leisure activity using open-ended questions, which were later categorized in three groups, it is possible that some of those who reported not having one considered that a leisure activity was limited to certain types of activities (e.g. practicing a sport or playing a musical instrument), and did not consider other regular activities, such as reading or watching TV, as a leisure one. However, if that was the case, we might assume participants did not see these activities as relaxing, rewarding, or pleasant enough to be considered leisure activities. Finally, the lower rates of stress in the group of participants with leisure activities might be associated with other elements not considered in this study, such as personality traits. Similarly, as this was a cross-sectional study, it was not possible to establish cause-and-effect relationships between engagement in leisure activities and participants' stress, perceived health, and overall wellbeing.

Despite its limitations, this study has several strengths that need to be highlighted. First, to our knowledge, this is the first study looking into the impact of leisure activities on psychiatrists' and trainees' wellbeing, thus providing novel evidence regarding the role and benefits of such activities for these professionals. Second, this study explored other elements of health and wellbeing, including participants' self-perception of their health and stress. Although the answers were in a self-reported format, their inclusion allowed us to explore of the different mechanisms by which leisure activities might impact individuals, even if these elements were not addressed in detail. Third, as this study included both, psychiatrists and psychiatry trainees, we were able to identify differences regarding their engagement in leisure activities, and even when these differences were not significant, we identified that trainees were less likely to have a leisure activity. This might not only be a reflection of their workload and lack of spare time, known social determinants of health, but it might also represent an opportunity to encourage the adoption of leisure activities for trainees as an intervention to improve their wellbeing. Finally, with this study, we provide some evidence supporting the importance of also studying and addressing other social determinants of health, specifically those which can positively impact people's wellbeing and physical and mental health.

### Implications of the results for future practice and research

Overall, this study suggests that Mexican psychiatrists' and trainees' wellbeing benefits from engaging in leisure activities, as even if none of the leisure activities were associated with better self-perceived health, all of them, regardless of type, were associated with lower perceived stress, improved confidence in stress management, and better satisfaction with social support. Results from this study also suggest that encouraging the adoption of leisure activities in psychiatrists might help improve some social determinants of health and wellbeing, especially during a time when workload, mental health problems and burnout have reached alarming peaks among healthcare workers (including mental health professionals) due to the COVID-19 pandemic [see for example, in Mexico: (37)]. Still, it is important to conduct further studies with more methodological rigor to assess the impact of having a leisure activity on doctors' levels of burnout, and to identify if any activity is more likely to positively impact professionals' wellbeing, as this could allow to design interventions which can address these social determinants of health.

### Conclusions

We conclude that some Mexican psychiatrists and psychiatry trainees already benefit from engaging in some form of leisure activity. However, we consider this should be further promoted in this population, as an easy-to-implement intervention to improve psychiatrists' and psychiatry trainees' wellbeing and mental health.

## Data availability statement

The datasets presented in this article are not readily available as they contain additional information - clinical and demographic - to that presented in this article. Therefore, requests to access the datasets should be directed to the corresponding author.

## Ethics statement

The studies involving human participants were reviewed and approved by Ethics and Research Committees of the Ramón de la Fuente Muñiz National Institute of Psychiatry in Mexico City (No. 09-CEI-010-20170316). All participants consented to take part in the study through an online survey.

## Author contributions

AF and EL-C conceptualized the research. TH and DT performed data curation. AF and CT-Z performed formal analysis. MY-N, DG-S, and RR-G performed investigation, methodology, and logistics. AF and RS-A supervised the development of the research. All authors contributed to the writing, editing and review of the manuscript. All authors approved the submitted version.
